# The impact of photodynamic therapy on immune system in cancer – an update

**DOI:** 10.3389/fimmu.2024.1335920

**Published:** 2024-02-28

**Authors:** Tomasz Dudzik, Igor Domański, Sebastian Makuch

**Affiliations:** ^1^ Faculty of Medicine, Wroclaw Medical University, Wroclaw, Poland; ^2^ Department of Clinical and Experimental Pathology, Wroclaw Medical University, Wroclaw, Poland

**Keywords:** photodynamic therapy, PDT, immune system, clinical studies, photosensitizers, cancer

## Abstract

Photodynamic therapy (PDT) is a therapeutic approach that has gained significant attention in recent years with its promising impact on the immune system. Recent studies have shown that PDT can modulate both the innate and adaptive arms of the immune system. Currently, numerous clinical trials are underway to investigate the effectiveness of this method in treating various types of cancer, as well as to evaluate the impact of PDT on immune system in cancer treatment. Notably, clinical studies have demonstrated the recruitment and activation of immune cells, including neutrophils, macrophages, and dendritic cells, at the treatment site following PDT. Moreover, combination approaches involving PDT and immunotherapy have also been explored in clinical trials. Despite significant advancements in its technological and clinical development, further studies are needed to fully uncover the mechanisms underlying immune activation by PDT. The main objective of this review is to comprehensively summarize and discuss both ongoing and completed studies that evaluate the impact of PDT of cancer on immune response.

## Introduction

Photodynamic therapy (PDT) is a treatment method that involves the use of a photosensitizer, an appropriate wavelength, and oxygen to induce targeted cell death (1). Originally developed in Germany about a century ago, PDT has evolved over the years and is now used in dermatology and as part of cancer therapy (2). The main purpose of PDT is to selectively destroy pathological tissues. This involves the use of a light-sensitive compound, known as a photosensitizer (PS) that accumulates in appropriate tissues. Once the photosensitizer has accumulated, an appropriate wavelength is applied to the target area, resulting in selective destruction of the targeted cells. In the presence of oxygen, a sequence of events occurs, leading to the direct death of tumor cells, damage to the microvasculature, and induction of a local inflammatory reaction (3).

Technological advances have allowed for the development of newer photosensitizers that minimize side effects and reduce normal tissue toxicity. Furthermore, nanotechnology has enabled the targeting of specific receptors, thereby increasing the selectivity of PDT (4). This treatment option is associated with minimal normal tissue toxicity, negligible systemic effects, reduced long-term morbidity, lack of intrinsic or acquired resistance mechanisms, and excellent cosmetic as well as organ function-sparing effects. Two groups of new PS can be distinguished. The first group comprises oxygen-carrying nanosystems, such as oxygen-carrying nanobubbles and nanodroplets, perfluorocarbon-based O_2_ nanocarrier, or hemoglobin-polymer conjugates as nanocarriers. The second group consists of oxygen-generating nanosystems like MnO_2_ nanoparticles, nonfluorinated chitosan-chlorine6/catalase nanoparticles, or biomimetic nanothylakoids (5). As a result, PDT is increasingly being considered as an important therapeutic option for combination treatments (3). In addition, recent research shows that well-known molecules may have undiscovered properties. Temozolomide (TMZ), generally known as a chemotherapeutic drug with efficacy for glioblastoma, can produce reactive oxygen species under the influence of ultrasound (6).

One of the promising applications of PDT is its use in controlling microbial biofilms, which are often associated with antibiotic resistance. PDT has shown reliable and realistic results in treating conditions such as oral caries and dental plaque, chronic wound infections, infected diabetic foot ulcers, cystic fibrosis, chronic sinusitis, and implant implant-associated infections (7).

This review explores how PDT affects immune cells, the potential benefits of combining tumor PDT with immunotherapy, the use of Immuno vaccines with PDT, the impact of combining Immune adjuvants with PDT, the role of PDT in targeting immune checkpoints, and the various effects of photosensitizers on the immune system in cancer treatment. Additionally, our focus extends to a thorough examination of ongoing clinical trials evaluating the impact of photosensitizers on immune response following PDT in cancer therapy. The main purpose of this review is to summarize and analyze both ongoing and completed studies that evaluate the impact of PDT of cancer on immune response.

## Influence of PDT on immune cells

The effect of PDT on immune cells is the third most important factor besides direct cell destruction and damage to the vascular skeleton of the tumor (8). Unlike the first two, its effect is time-shifted, but the effect that PDT has on the cells of the immune system, makes it possible to achieve long-term effects not only in the primary tumor focus, but also on the whole body and indirectly on metastasis (9). It is postulated by researchers that neutrophils play a pivotal role in the context of tumor PDT, thereby signifying their indispensability in this therapeutic approach (10).

Neutrophils, as the largest group of immune cells, contribute the most to the first stage of the immune response after PDT with the photosensitiser methylene blue (MB). It has been noted that neutrophils have increased cell adhesion, but their myeloperoxidase activity is not altered, while at the same time increasing the production of reactive oxygen species, crucial in the destruction of cancer cells. Interestingly, a reduced fungicidal capacity has also been noted (11). Furthermore, it has been observed that PDT elicits an upregulation in the expression levels of adhesion molecules, including E-selectin and ICAM1. These crucial molecules facilitate the adhesion of circulating cells to tumor microvessels, subsequently facilitating their extravasation and infiltration into the tumor tissue (12, 13). Moreover, this process also triggers the activation of the complement system, which is known to be activated by PDT (14). Notably, PDT induces a robust acute-phase response, characterized by increased levels of C-reactive protein (CRP), mannose-binding lectins (MBL), and serum amyloid P (SAP). These proteins play a crucial role in facilitating the recruitment and activation of neutrophils, thereby augmenting the overall immune response within the tumor microenvironment (15). Additionally, it is noteworthy that lymph nodes also play a significant role in this process. Neutrophils have been observed to infiltrate lymph nodes that are burdened with tumor cells, and this phenomenon is facilitated by the IL-17 and IL-1β MIP2-mediated pathway (16).

**Figure 1:**
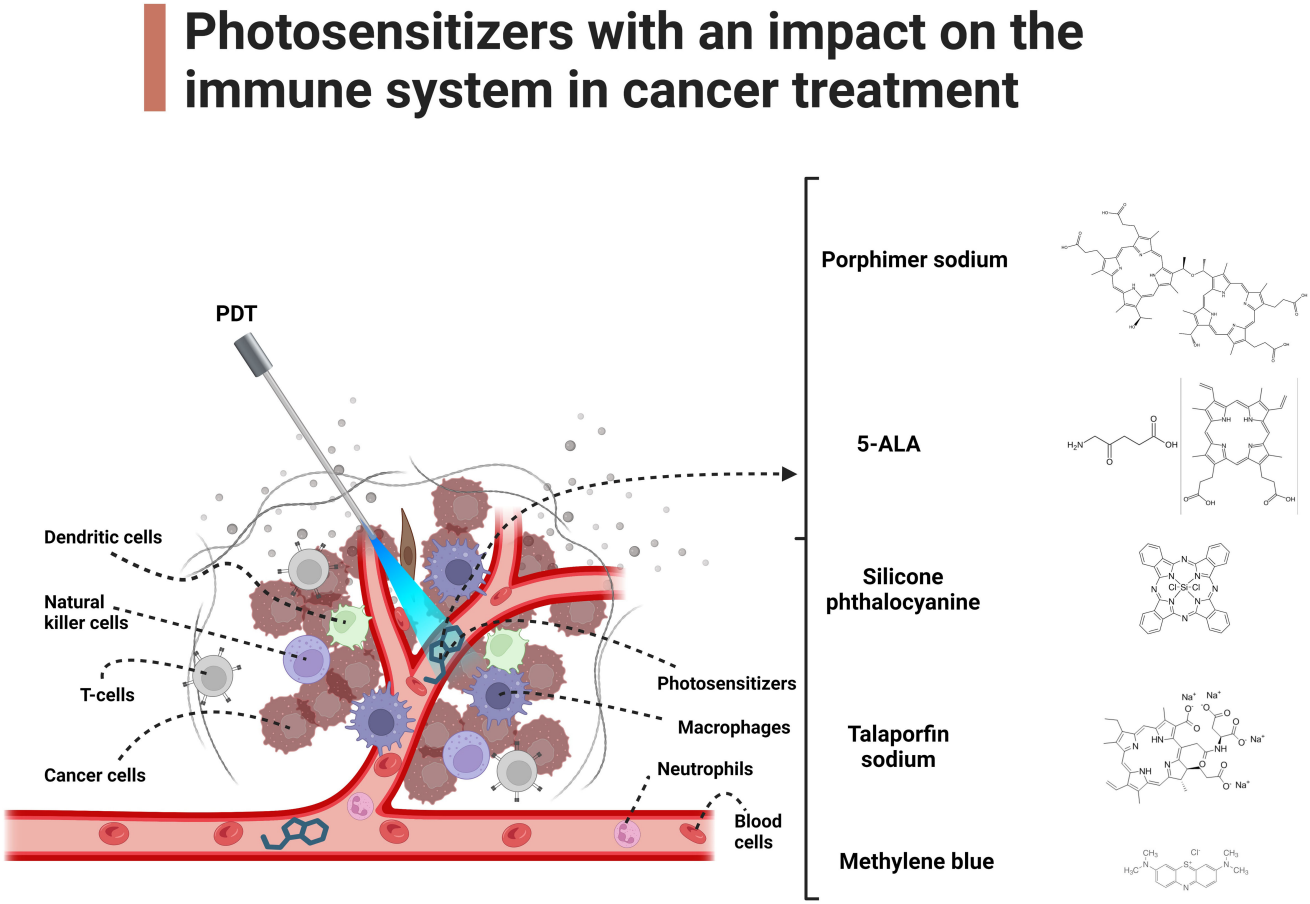
Photosensitizers with an impact on the immune system in cancer treatment. The main text provides a detailed exploration of the molecular mechanisms underlying the impact of PDT on immune cells in cancer.

Macrophages are characterized by the expression of complement receptors, which equip them with the capability to phagocytize tumor cells opsonized with C3 and mannose-binding lectins (MBLs) (17). In addition, PDT induces the release of heat shock protein 70 (Hsp70), a damage-associated molecular pattern (DAMP) that binds to Toll-like receptors 2 and 4 (TLR2/4) on tumor cells. This event triggers the activation of macrophages and subsequent release of tumor necrosis factor alpha (TNF-α) (18). After PDT, macrophages together with neutrophils infiltrate the tumor with a large release of inflammatory factors within the tumor (19). This is very important due to the same initiation of a cellular, more specific immune response. In addition, it has been found that with the use of the photosensitizer 5-aminolevulinic acid (ALA), the differentiation of M1/M2 macrophages and an overall increase in their number is observed (20). Photosensitizers with an impact on the immune system in cancer treatment has been illustrated in **Figure 1**.

PDT further leads to an increase in specific immune system responses. PDT exerts an influence on the expression of major histocompatibility complex class I-like molecules and NKG2D ligands on cells subjected to treatment, as evidenced by studies. These molecular alterations enable natural killer (NK) cells to actively participate in the immune process (21, 22). An overall increase in the number of NK cells has been observed, not only within the tumor and surrounding tissues, but also in the peripheral blood (23). In addition, NK cell activity increased, as determined by an increase in TNF-α release (24). Notably, other investigations have proposed an indirect mechanism of action for NK cells. Specifically, splenic NK cells isolated from mice subjected to PDT treatment exhibited noncytotoxic effects on cells *in vitro* (25). Further observations noted a stimulation of T cells (both CD4+ and CD8+) (26), as well as an increase in their number (8). They induce the destruction of tumor cells and are considered the most important element of the immunological part of PDT in cancer treatment. Without them, the immunological effect of PDT has been shown to be reduced and, in some cases, abolished (27, 28).

Studies have revealed that the transfer of CD4+ T cells derived from PDT-treated mice resulted in reduced or delayed tumor growth when introduced into naive mice (29). Moreover, it has been documented that both CD4^+^ and CD8^+^ T cells play crucial roles in the immune processes associated with PDT (27). Notably, Th17 cells have emerged as important contributors to neutrophil activation (30). Furthermore, an increased number of Th17 cells and the role of interleukin-17 (IL-17) in the recruitment of neutrophils have been reported (16). Collectively, these findings emphasize the intricate interplay between various immune cell subsets, such as CD4^+^ and CD8^+^ T cells, as well as the significant involvement of Th17 cells and IL-17 in the immune response accompanying PDT.

## Tumor photodynamic therapy combined with immunotherapy

Immunotherapy and PDT have some limitations and are not able to meet the demands of the current cancer treatment. PDT is low invasive and has a limited amount of side effects and on the other hand immunotherapy has significant clinical applications in the treatment of cancer. Combining those two therapies may improve the efficacy of those treatment methods (31).

Immunotherapy is a cancer therapy, which specifically targets cancer cells by immune cells and other molecules. The great advantage is long term effect without harm to non-cancerous cells (32, 33). Today we are able to offer different immune methods of treatment, such as cancer vaccines, adoptive cellular immunotherapy or immune checkpoint blockade (34). Worth of mention is that not every patient can use this treatment. High amount of side effect such as rashes, itching, diarrhoea, pneumonia, hematopoietic system dysfunction, thyroid malfunction and organ toxicity limits the usage of the treatment (35, 36).

PDT can trigger the immune system by releasing tumor antigens and promoting the maturity of dendritic cells, while immunotherapy can enhance the antitumor immune response and overcome the immunosuppressive environment in tumors. Recent studies by (37). found that the combination of PDT with immune checkpoint inhibitors and TLR7 agonists, respectively, demonstrated significant improvements in treating melanoma and breast cancer in mouse models.

The administration of PDT to animals leads to the production of a serum that displays immunological adaptations. This serum is capable of eliciting an immune response that effectively impedes the progression of homologous transplantation tumors in the animal model (38, 39).

The successful treatment of bilateral melanoma in mice has been demonstrated through the utilization of a combination of PDT and Toll-Like Receptor 5 (TLR5) agonist flagellin. This approach has been shown to not only effectively suppress tumor growth in the tumor microenvironment (TME), but also to enhance the presentation of tumor antigens via cross-presentation. Additionally, the use of PDT and TLR5 agonist flagellin has been shown to stimulate the infiltration of tumor CD8+ T cells and the secretion of Interferon-gamma (IFN-γ) throughout the entire organism (40).

It is worth noting that the novel modality of laser immunotherapy (LIT) has recently been developed, which combines the utilization of PDT with an immunological adjuvant. The primary components of LIT are Indocyanine green (ICG) and glycated chitosan (GC), which have been shown to induce personalized, tumor-specific immunity. In recent studies, LIT has demonstrated superior therapeutic efficacy compared to PDT alone, with GC exhibiting stronger immune-stimulatory abilities than other commonly used adjuvants (41). Furthermore, while PDT vaccines have been associated with a significant increase in the levels of immunosuppressive cells, such as myeloid-derived suppressor cells and regulatory T cells, the concomitant administration of low doses of GC and cyclophosphamide has been shown to effectively mitigate this elevation (42). Kim et al. found that by clearing local immunogenicity, the tumor microenvironment was reconstructed from a cold to hot state, leading to an increase in cytotoxic CD8^+^ T cells and a spread of systemic anti-tumor immunity. This resulted in an extended survival time and highlights the potential of sensitizing the PD-1 or PD-L1 immune checkpoint blocking reaction (43).

## Immuno vaccines combined with PDT

One of the most encouraging forms of combination therapy involves utilizing cancerous cells treated with PDT as a vaccine for dendritic cells (DCs). Research has demonstrated that such vaccinations can elicit a highly potent immune response (44). One of the famous researchers in this field Korbelik has made a vaccine against squamous cell carcinoma (SCCVII) using PDT. He showed inhibition of tumor growth using this vaccine (42).

Inducing immunogenic cell death (ICD) through Endoplasmic Reticulum (ER)-targeting PDT with Par-ICG-Lipo loaded with indocyanine green (ICG) has been found to enhance the immunogenicity of *in situ* tumor cells *in vivo*, which, when combined with dendritic cells, could provide an effective and clinically applicable approach for cancer treatment by transforming cancer tissue into a therapeutic tumor vaccine (45).

Another study aims to develop patient-adjusted immunotherapies for gliomas, the most common type of primary tumor of the central nervous system in adults. The researchers investigated the efficacy of dendritic cell vaccines loaded with glioma cells undergoing immunogenic cell death induced by photosens-based PDT and identified a four-gene signature associated with overall survival of glioma patients. The results suggest that this approach has the potential to improve glioma therapy by inducing Th17 immunity and predicting patient outcomes (46).

Other researchers decided to use amphipathic 4T1 breast cancer membrane to support loading Ce6 (chlorin e6) as PS and Dox (doxorubicin hydrochloride). The membrane was coated by calcium carbonate to construct a nano drug delivery system. Result was that simultaneous influence of PTD and Dox caused tumor immunogenic cell death and releasing tumor associated antigens. Expected is that Reactive Oxygen Species (ROS) generated thank to this process will form PDT-DC vaccination by mimicking inflammatory mechanisms to recruit DCs (47).

## Immune adjuvants combined with PDT

PDT was also tested with immune adjuvants. One of the most promising is imiquimod. Imiquimod is a TLR7 agonist and it’s registered by FDA as a treatment for various skin diseases (48). Imiquimod interacts with TLR7 on the DC and endosomes, what leads to DC’s maturation and releasing pro-inflammatory cytokines (49). The usage of imiquimod in cream with PTD supported 5-aminolevulinic acid as a PS succeed in treatment of squamous cell carcinoma of the skin (50).

MPSNs (mesoporous hexagonal core-shell zinc porphyrin-silica nanoparticles) have the potential to not only function as exceptional photosensitizers for photo-immunotherapy, but also as an optimal drug carrier for achieving more effective synergy, wherein MPSNs loaded with R837 can promote immunogenic cell death via PDT and Photothermal Therapy (PTT), and induce tumor-specific immune responses by facilitating dendritic cell maturation after the pH-responsive release of R837, ultimately resulting in significant inhibition of primary and metastatic tumors with minimal systemic toxicity when combined with programmed death ligand-1 checkpoint blockade, highlighting the promising therapeutic approach that combines PTT, PDT, and checkpoint blockade for suppressing cancer metastasis (51).

The synthetic dipeptide molecule, Pidotimod, has been shown to enhance immune responses and protect against infection in mice and humans, but its exact mechanism of protection is unclear; a study using zebrafish models found that while PDT increased recruitment of immune cells and promoted pro-inflammatory cytokine production in the tail wound assay, it did not provide protection against infection with certain pathogens (52).

It was shown that PDT causes inflammation and immunosuppression due to contact hypersensitivity. It is important to find an immune inhibitor to soften Tumor immunosuppressive signals and enable PDT to induce full immune response (53–55).

## PDT targeting immune checkpoints

Immunotherapy also includes treatments targeting immune checkpoints, which regulate the immune system and can inhibit the immune response against cancer cells. Those checkpoints have negative immunomodulatory effect (56).

Tumor burden serves as a useful biomarker to guide the use of immune-checkpoint inhibitors, and future therapeutic strategies can address the issue of inferior outcomes among patients with cancer and high tumor burden (57). Modern treatments use antibodies which are target-specific to block various immune checkpoints (58). PDT is able to assist in this process by enhancing tumor immunogenicity and sensitivity by introducing ICD (59, 60). Combining FIC-PDT (Ce6-embedded nanophotosensitizer) with ripasudil induces immunogenic cell death and stimulates antigen-presenting cells to prime tumor-specific cytotoxic T cells, sensitizing the response to PD-1/PD-L1 immune checkpoint blockade and resulting in potent antitumor effects in an intraocular melanoma model (43). PDT has been suggested as a potential complementary strategy for immune checkpoint inhibitors, such as CTLA-4, PD-1/PD-L1, and CD47 targeted treatment. This approach is based on the ability of PDT to stimulate the immune system and enhance the antitumor response triggered by immune checkpoint inhibitors. Several studies have shown promising results in combining PDT with immune checkpoint inhibitors, suggesting a potential for improving the efficacy of cancer immunotherapy (61–63).

Clinical studies on the effects of PDT on the human immune system are limited, whereas animal models have been used extensively to develop and test various PSs. The lack of clinical studies highlights the need for more research to explore the potential of PDT in human cancer therapy. Despite this, the positive results observed in animal studies provide a promising foundation for further investigation into PDT and its potential benefits in clinical settings (31).

## Photosensitizers with an impact on immune system in cancer treatment

PDT has the ability to modulate the immune system, enabling it to identify and eliminate cancer cells effectively. Numerous studies using different photosensitizing agents have shown the immunomodulatory properties of PDT.

Talaporfin sodium (TS) treatment has been found to increase calreticulin (CRT) expression on the plasma membrane and translocate CRT from the nucleus to the cytoplasm, as observed through immunofluorescence staining (64). Moreover, TS-PDT induced the release and/or expression of DAMPs, indicating activation of innate immunity (64). In another study, significantly higher levels of IL-2 and IFN-γ, along with lower levels of IL-10 were observed in mice treated with Haematoporphyrin derivative (HpD) and ultrasound irradiation on day 10 after continuous irradiation (65). These findings suggest that the application of SDT (sonodynamic therapy) along with HpD could stimulate innate immune responses, trigger inflammation, and promote a shift from Th2 to Th1 cells within tumors (65). Another photosensitizer, methylene blue (MB) has been shown to induce THP-1 macrophage apoptosis *in vitro* and alleviate periodontitis in rats (66). Moreover, MB-PDT increases human neutrophil adhesion and does not modify myeloperoxidase release (11).

PDT using mTHPC combined with PD-L1 blockade demonstrated long-lasting immune memory response, preventing tumor recurrence. This combination therapy effectively suppressed tumor growth, including non-irradiated distant tumors, through a potent and synergistic anti-tumor immune response (67). Novel nanocomposite called TiO2@Ru@siRNA, which consists of ruthenium-based photosensitizer (Ru) modified-TiO2 nanoparticles (NPs) loaded with siRNA targeting hypoxia-inducible factor-1α (HIF-1α), demonstrated effective therapeutic outcomes and prognostic implications for oral squamous cell carcinoma (OSCC) by modulating the hypoxic and immune microenvironment (68). Upon visible light irradiation, TiO2@Ru@siRNA induced both Type I and Type II photodynamic effects, resulting in lysosomal damage, efficient silencing of the HIF-1α gene, and elimination of OSCC cells. By alleviating hypoxia and inducing pyroptosis, TiO2@Ru@siRNA remodels the immune microenvironment by reducing key immunosuppressive factors, increasing immune cytokine expression, and activating CD4+ and CD8+ T lymphocytes. Promisingly, in patient-derived xenograft (PDX) and rat oral experimental carcinogenesis models, TiO2@Ru@siRNA-mediated PDT significantly suppresses tumor growth and progression while enhancing cancer immunity (68).

In contrast, Indocyanine green (ICG) is a versatile photosensitizer that exhibits sensitivity to a broad range of wavelengths, particularly in the near-infrared (NIR) region, with peak absorption in this range. It possesses excellent excretion properties and can generate singlet oxygen, which effectively damages cancer cells by reacting with various intracellular macromolecules such as nucleic acids, proteins, and lipids. Consequently, singlet oxygen-induced cell injury leads to endoplasmic reticulum stress, triggering immunogenic cell death (ICD) and the release of damage-associated molecular patterns, including calreticulin and high-mobility group box-1. Additionally, ICG has the potential to induce an antitumor response through photothermal therapy (PTT) as it produces heat upon NIR irradiation. However, rapid metabolism after intravenous administration limits ICG accumulation in cancerous tissues. To overcome this, recent studies have explored the use of ICG encapsulated within liposomes or micelles, enabling enhanced permeability and retention (EPR) effect-mediated accumulation in cancer cells LEM has shown potential in suppressing tumor growth by alleviating regulatory T cell-mediated immunosuppression, while hydrogen gas inhalation therapy enhances the efficacy of immune checkpoint inhibitors. The combination of these therapies with PDT using ICG liposomes offers a simple and cost-effective treatment approach that can boost the body’s immune response against tumors without burdening patients with side effects (69).

## Clinical trials evaluating the impact of photosensitizers on immune response following PDT in cancer treatment

### Porfirmer sodium

Utilizing porfimer sodium in the context of non-small cell lung cancers, combined with light-based interventions during surgical procedures, exhibited alterations in the immune phenotype of peripheral blood CD8+ T cells and modifications in the platelet-to-lymphocyte ratio subsequent to a single PDT session. The activation of CD8+ T cells primarily relies on the maturation and activation of dendritic cells. Activated dendritic cells facilitate the differentiation and activation of T cells by expressing antigenic peptide: MHC complexes, costimulatory molecules, and cytokines, thereby eliciting the development of antitumor immune responses. PDT leads to the maturation and activation of dendritic cells, prompting their migration to the adjacent lymph nodes, where they are presumed to induce T-cell activation (70, 71).

The induction of acute inflammatory response following PDT in the tumor stroma is a consequence of changes in tissue integrity and homeostasis, resulting in cell necrosis and oxidative damage. This process is initiated by the release of pro-inflammatory mediators such as cytokines, growth factors, and proteins. At the site of injury, innate immune cells including neutrophils, mast cells, macrophages, and dendritic cells participate in the phagocytosis of cancer cell breakdown products and contribute proteins to helper CD4+ T lymphocytes. Cytotoxic T cells, on the other hand, possess the ability to recognize and specifically eliminate tumor cells, thereby generating a systemic anti-tumor immune response that can persist throughout the body for an extended duration. With successive cycles of absorption, the photosensitizer (PS) may degrade, losing its capacity to trigger a photodynamic reaction. This phenomenon is known as photobleaching, signifying the process of PS burnout (72).

Currently, there are only a few ongoing clinical trials investigating the use of porfimer sodium. One of these trials is being conducted on patients with non-small cell lung cancer. The main objective of this study is to determine the incidence of serious adverse events (SAEs) by closely monitoring and documenting any occurrences during the first 28 days following the administration of study-related therapy (NCT04836429) (73).

Promising outcomes have been observed in the use of porfimer sodium for non-small cell lung cancer. One study conducted on 10 patients showed positive responses to PDT in all cases, with improvements in physical airway obstruction, resolution of acute hemoptysis, and a median survival of 5.5 months post-PDT. Three patients were alive at the time of study evaluating, ranging from 5 to 21 months after therapy. PDT offers effective relief for hemoptysis, dyspnea, and airway obstruction, enhancing the patients’ quality of life (74).

Also porfimer sodium demonstrated a statistically similar delay in tumor regrowth after PDT when treating small tumors at 24 and 48 hours. However, at 72 hours, porfimer sodium performed better than PS785 in delaying regrowth of small tumors. For large tumors, porfimer sodium did not exhibit any significant delay in tumor regrowth at any of the time points studied (75).

A separate study is currently underway, encompassing various diseases including locally advanced lung carcinoma, non-small cell lung carcinoma, and small cell lung carcinoma in stage III. The primary objectives of this trial are to assess the incidence of grade 4 or higher adverse events, according to the Cancer Institute Common Terminology Criteria for Adverse Events. Additionally, the study aims to determine the tumor response rate, which will be reported using frequencies and relative frequencies. Furthermore, progression-free survival will be evaluated using the standardized Kaplan-Meier methods (NCT03735095).

Another clinical trial is currently being conducted to investigate malignant mesothelioma, non-small cell lung carcinoma, and pleural disorders. The primary objectives of this trial are to determine the incidence of adverse events and assess toxicity levels associated with the treatment. The researchers will systematically record and tabulate the adverse events according to their respective grades across all dose levels and treatment cycles. Moreover, progression-free survival will be analyzed using the Kaplan-Meier method, while overall survival outcomes will also be obtained. In addition, the trial aims to monitor anti-tumor responses by evaluating immune markers and detecting any instances of local or distant disease recurrence (NCT03678350).

A separate study focuses on recurrent head and neck carcinoma or locally advanced head and neck carcinoma. The primary objective of this study is to determine the incidence of adverse events and assess the frequency of toxicities, which will be systematically tabulated by grade across all dose levels and treatment cycles. Sequential boundaries will be employed to monitor any occurrence of serious adverse events. Notably, researchers will compare the efficacy of treatment using I-PDT followed by standard of care against standard of care alone. The primary analysis will involve the use of Fisher’s exact test to evaluate objective tumor response rates at 10-12 weeks. It is important to note that the primary analysis will be conducted based on the intention-to-treat principle (NCT03727061) (76).

One study aimed to assess the effectiveness of porfimer sodium-mediated PDT in patients diagnosed with head and neck squamous cell carcinoma. The results indicate that PDT is a valuable treatment option for managing head and neck squamous cell carcinomas, leading to enhanced quality of life, particularly in cases of recurrent or residual disease (77). Also another study examining patients diagnosed with carcinoma-*in-situ* and T1 carcinomas achieved complete response following a single PDT treatment. The majority of these patients have maintained disease-free status, with only two exceptions. In a separate group of ten patients with extensive neck recurrences of squamous cell carcinomas, intraoperative adjuvant PDT was administered following tumor resection. With a follow-up period of 40 months, only three patients experienced recurrence, and notably, only one recurrence occurred within the surgical and PDT-treated area (78).

### Silicon phthalocyanine

Pc 4 PDT-induced immunosuppression involves the contribution of both CD8+ and CD4+ T cells (79). Pc 4 uptake was found to be higher in T cells associated with cutaneous T cell lymphoma (CTCL) than in normal resting T cells. Pc 4-PDT treatment resulted in a stronger apoptotic effect on activated CD3(+) T cells, suggesting a potential therapeutic strategy for skin disorders mediated by T cells, including CTCL. Interestingly, among normal human T cells, it is observed that activated T cells with many mitochondria had the highest Pc 4 uptake, followed by resting T cells, while Treg cells had the lowest uptake. This suggests that Pc 4-PDT could be particularly effective in targeting reactive T cells in chronic inflammatory conditions or neoplastic T cells in T cell lymphomas/leukemias. There may be a therapeutic window where resting naïve and memory T cells survive, along with the preservation of Treg cells that can restore quiescence once the population of auto-reactive inflammatory cells decreases (80). According to a study, the use of Pc 4 applied topically shows promise in the treatment of cutaneous T-cell lymphoma (CTCL) and potentially other skin cancers such as basal cell carcinoma. Additionally, Pc 4 demonstrates potential for managing non-malignant skin conditions like psoriasis. Moreover, when Pc 4 is formulated in an appropriate vehicle, it has the potential to be effectively delivered to accessible cancers in areas like the head and neck, esophagus, and lung (81).

### 5-ALA

Over thirty years ago, the use of 5-aminolevulinic acid-based PDT (ALA-PDT) was introduced and has since become an integral part of clinical practice for the treatment of both pre-cancerous and cancerous skin lesions.

Since the effects of ALA are well understood in the context of its use as a therapy against BCC, research is underway on an ethylated ALA equivalent known as MAL. In this study, MAL therapy was proven to be more effective than ALA. It is worth to say that the cosmetic outcome was similar in both therapies (82). However, another study that compares the histological clearance, tolerability and cosmetic outcome of MAL, BF-200 ALA, and low-concentration Hexyl aminolevulinate (HAL) in the PDT of non aggressive BCC. The conclusion of this article is that each method has a similar effect, but with using HAL this outcome is achieved with low concentration (83).

The aims of one on the present trials is to determine time to maximum expression of immune checkpoint molecules in BCC tumors and peri-tumoral stroma after PDT, as compared to untreated tumors. Moreover, to determine the ratio of cytotoxic T cells to regulatory T cells in BCC tumors and peri-tumoral stroma after PDT, as compared to untreated tumors, specific antibodies against the following markers were used to measure the infiltration of different immune cell populations: Neutrophils (Gr1+ or MPO+), Macrophages (F4/80+), MDSCs (CD33, S100A9), cytotoxic T cells (CD8+), regulatory T cells (CD4+, FoxP3+, CD25+, CD127-), and NK cells (CD56+ CD16+) (NCT05020912).

The use of topical 5-aminolevulinic acid mediated PDT (ALA-PDT) can increase the expression of damage-associated molecular patterns (DAMPs) such as CRT, HSP70, and HMGB1. This promotes the activation of dendritic cells (DCs) and stimulates anti-tumor immune responses. The process involves the maturation of DCs in both phenotype and function, including an increase in the surface expression of MHC-II, CD80, CD86, as well as an enhanced ability to secrete IFN-γ and IL-12 (84). Celecoxib is an anti-inflammatory drug that induces intracellular ROS generation (85). It is known that these two molecules have synergistic effects.

Another study aims to determine what percentage of patients affected by Pleural Mesotheliomas or Malignant Pleural Mesothelioma will respond to combined treatment with ALA-PDT followed by adjuvant immunotherapy with anti-PD-1 Nivolumab antibodies while maintaining low treatment toxicity (grade≥3) according to National Cancer Institute (NCI) criteria (NCT04400539).

Another cancer against which PDT using ALA is being used is glioblastoma. FDA in 2017 approved 5-ALA for glioma surgery (86). The objective of surgery for individuals with Glioblastoma Multiforme (GBM) is to achieve complete removal of the tumor while avoiding any new neurological deficits. However, the process of surgical resection is frequently limited because it is challenging to distinguish the tumor from the surrounding healthy brain tissue using conventional methods such as visual examination or standard intraoperative white light microscopy. To overcome this limitation, orally administered 5-ALA has been employed to enhance the identification of the tumor during the surgery. When viewed under a specific light, GBM cells that have taken up 5-ALA become fluorescent, allowing for improved intraoperative tumor detection (87). Current ongoing research focuses on the incidence of disorderly actions. Immunological parameters are also being analyzed when using this therapy (PBMC, CD4+, CD8+) (NCT03897491). Ongoing clinical trials that evaluate the impact of PDT of cancer on immune response have been summarized in **Table 1**.

**Table 1:** Ongoing clinical trials of photosensitizers in cancer treatment with a potential impact on immune response.

Diseases	PS	Illumination Protocol	Potential Immune Response	Clinical trial ID
Non-small cell Lung cancer	Porfimer Sodium	One course of light therapy at the time of surgery	Changes in the immune phenotype of peripheral blood CD8+ T cells/Changes in platelet-to-lymphocyte ratio	NCT04836429 (73)
Basal Cell Carcinoma	5-ALA	Blue light at first visit for 30 mins/20 mJ/cm^2^	Determination of the time to maximum expression of immune checkpoint molecules in BCC tumors and peri-tumoral stroma after PDT, and also the ratio of cytotoxic T cells to regulatory T cells in BCC tumors and peri-tumoral stroma after PDT, as compared to untreated tumors	NCT05020912 (88)
Locally Advanced Lung Carcinoma; Non-Small Cell Lung Carcinoma; Small Cell Lung Carcinoma	Porfimer Sodium	I-PDT undergo Endobronchial Ultrasound with Transbronchial Needle(EBUS-TBN) over 30-45 minutes	Examine Porfimer sodium retention in the target tumor tissue and the relationship between immune biomarkers and response	NCT03735095 (89)
Malignant Mesothelioma; Non-Small Cell Lung Carcinoma; Pleural Disorder	Porfimer Sodium	IO-PDT via a light dosimeter your system 24-28 after taking PS	Monitor immune markers for correlation between the IO-PDT treatments and local or distant disease recurrence	NCT03678350 (90)
Recurrent Head and Neck Carcinoma; Locally Advanced Head and Neck Carcinoma	Porfimer Sodium	Patients receive Porfimer sodium IV over 3-5 minutes and undergo I-PDT approximately 48 hours later	Evaluation of the relationship between response rate and immune markers in patients with locally advanced or recurrent HNC receiving either adjuvant porfimer sodium mediated I-PDT	NCT03727061 (76)
Malignant Pleural Mesothelioma	5-ALA	20 mg/kg; 400-500 nm; 25 J/cm^2^) during 15 minutes (6 fractions of 2.5 minutes separated by 5 pauses of 2 minutes each to improve tissue oxygenation for the PDT reaction	Demonstration of the feasibility of combining intrapleural PDT and immunotherapy by Nivolumab	NCT04400539 (91)
Glioblastoma	5-ALA	20 mg/kg orally four hours before anaesthesia; 200 mW/cm diffusor length	Analytical results for immune parameters (PBMC, CD4+, CD8+) in the respective blood samples of each patient	NCT03897491 (92)

## Conclusions

Photodynamic therapy represents a promising therapeutic approach suitable for patients who are deemed ineligible for surgical intervention. The mounting body of evidence highlights the pivotal role of the immune system in this therapeutic modality. The involvement of numerous complex mechanisms underscores the vast potential for further exploration and investigation of potential avenues to utilize this method in cancer treatment. While an increasing number of photosensitizers (PSs) are being utilized in research studies, the lack of a universally applicable PS capable of addressing a wide range of tumor types remains a challenge. Immunovaccines present a promising alternative to conventional chemotherapy, and their role in cancer treatment should be further emphasized. PDT offers a distinct advantage over radiotherapy and chemotherapy, as it is associated with minimal side effects. The ongoing clinical studies discussed in this paper aim to comprehensively evaluate and document the side effects observed in patients with diverse cancer types. The collection of such data and the expansion of our knowledge regarding immune mechanisms are paramount for future investigations among patients and for the incorporation of PDT into cancer treatment standards. Furthermore, additional studies are warranted to investigate various PSs and their impact on immune system dynamics. Our research has revealed a wide variety of PSs under investigation, yet there remains a lack of evidence regarding their effects on immune system cells.

## Author contributions

TD: Writing – original draft. ID: Writing – original draft. SM: Conceptualization, Writing – review & editing.
